# Is the COVID-19 Pandemic a Good Time to Include *Aspergillus* Molecular Detection to Categorize Aspergillosis in ICU Patients? A Monocentric Experience

**DOI:** 10.3390/jof6030105

**Published:** 2020-07-10

**Authors:** Jean-Pierre Gangneux, Florian Reizine, Hélène Guegan, Kieran Pinceaux, Pierre Le Balch, Emilie Prat, Romain Pelletier, Sorya Belaz, Mathieu Le Souhaitier, Yves Le Tulzo, Philippe Seguin, Mathieu Lederlin, Jean-Marc Tadié, Florence Robert-Gangneux

**Affiliations:** 1 Service de Parasitologie-Mycologie, CHU Rennes, F-35033 Rennes, France; helene.guegan@chu-rennes.fr (H.G.); emilie.prat@chu-rennes.fr (E.P.); romain.pelletier@chu-rennes.fr (R.P.); sorya.belaz@chu-rennes.fr (S.B.); florence.robert-gangneux@univ-rennes1.fr (F.R.-G.); 2 Irset (Institut de Recherche en Santé, Environnement et Travail)–UMR_S 1085, Univ Rennes, CHU Rennes, Inserm, EHESP, F-35000 Rennes, France; 3 Maladies Infectieuses et Réanimation Médicale, CHU Rennes, F-35033 Rennes, France; florian.reizine@chu-rennes.fr (F.R.); kieran.pinceaux@chu-rennes.fr (K.P.); pierre.le.balch@chu-rennes.fr (P.L.B.); mathieu.lesouhaitier@chu-rennes.fr (M.L.S.); yves.le.tulzo@chu-rennes.fr (Y.L.T.); jeanmarc.tadie@chu-rennes.fr (J.-M.T.); 4 Service de Réanimation Chirurgicale, CHU Rennes, F-35033 Rennes, France; philippe.seguin@chu-rennes.fr; 5 Service d’Imagerie Médicale, CHU Rennes, F-35033 Rennes, France; mathieu.lederlin@chu-rennes.fr

**Keywords:** invasive aspergillosis, putative, probable, COVID-19, Sars-CoV-2, ICU, PCR, *Aspergillus*, galactomannan, classification

## Abstract

(1) Background: The diagnosis of invasive aspergillosis (IA) in an intensive care unit (ICU) remains a challenge and the COVID-19 epidemic makes it even harder. Here, we evaluated *Aspergillus* PCR input to help classifying IA in SARS-CoV-2-infected patients. (2) Methods: 45 COVID-19 patients were prospectively monitored twice weekly for *Aspergillus* markers and anti-*Aspergillus* serology. We evaluated the concordance between (I) *Aspergillus* PCR and culture in respiratory samples, and (II) blood PCR and serum galactomannan. Patients were classified as putative/proven/colonized using AspICU algorithm and two other methods. (3) Results: The concordance of techniques applied on respiratory and blood samples was moderate (kappa = 0.58 and kappa = 0.63, respectively), with a higher sensitivity of PCR. According to AspICU, 9/45 patients were classified as putative IA. When incorporating PCR results, 15 were putative IA because they met all criteria, probably with a lack of specificity in the context of COVID-19. Using a modified AspICU algorithm, eight patients were classified as colonized and seven as putative IA. (4) Conclusion: An appreciation of the fungal burden using PCR and *Aspergillus* serology was added to propose a modified AspICU algorithm. This proof of concept seemed relevant, as it was in agreement with the outcome of patients, but will need validation in larger cohorts.

## 1. Introduction

Molecular tools as diagnostic criteria for invasive fungal diseases (IFD) has long been questioned because of a lack of reproducibility and insufficient standardization of protocols. Thanks to initiatives such as FPCRI (www.fpcri.eu [1]) and to the dramatic improvement of the quality assessment of molecular technics, *Aspergillus* PCR is now included in the new EORTC criteria for classification [2]. Regarding intensive care units (ICU) patients, the classification of IFD mainly refers on criteria adapted from neutropenic patients or relies on single center experiences. One algorithm has emerged as a valuable tool to classify invasive aspergillosis (IA) in ICU patients: the AspICU algorithm [3]. This classification is considered as robust because it has been evaluated in patients for whom autopsy results were available, but it is quite awkward to use in routine practice, particularly in COVID-19 patients with clinical and CT-scan signs hard to interpret [4]. Besides, it does not include molecular markers, which are now used routinely [5].

During COVID-19, patients presenting an acute respiratory distress syndrome (ARDS) shared risk factors and underlying diseases classically reported for IA, such as intubation and mechanical ventilation, corticosteroid therapy, immunological storm with high production of inflammatory cytokines. Warnings following preliminary cohort studies from various countries prompted the monitoring of fungal colonization and co-infections in SARS-CoV-2-infected patients hospitalized in an ICU. However, the entry criterion for putative IA, according to Blot et al., is an *Aspergillus*-positive culture endotracheal aspirate, which may lack specificity. In the recent review by Arastehfar et al. [6], many COVID-19-associated pulmonary aspergillosis (CAPA) benefited from galactomannan (GM) testing of bronchoalveolar fluid (BALF) or even of tracheal aspirates (not approved by the manufacturer). However, some laboratories, such as ours, have stopped various manipulations of highly SARS-CoV-2-infected samples in order to limit the exposure of laboratory technicians to viral infection. Then, direct examination of respiratory samples or galactomannan (GM) determination in broncho-alveolar lavage have thus been replaced by the systematic use of molecular tools. While performances of blood biomarkers such as GM, (1-3)β-d-glucan (BDG) or *Aspergillus* DNA detection are well evaluated in neutropenic patients, their clinical value is far less known in other conditions and still need evaluation in an ICU.

Here, our objective was to evaluate the concordance between molecular detection of *Aspergillus* in respiratory and culture and concordance between blood PCR and serum GM. We also aimed at assessing the ability of *Aspergillus* PCRs to help categorizing patients in the continuum of colonization to invasive infection in COVID-19 patients. Arguments to complement AspICU criteria are suggested.

## 2. Materials and Methods

### 2.1. Population of Patients

Forty-five intubated and mechanically ventilated patients hospitalized in a “COVID-19 ICU” of Rennes teaching hospital were screened for this study and benefited from a systematic monitoring to detect *Aspergillus*.

The hospital’s ethics committee (N 20-56 obtained the 30 April 2020) approved the study. The presence of SARS-CoV-2 in respiratory specimens (nasal and pharyngeal swabs or sputum) was detected by real time reverse transcription-polymerase chain reaction (RT-PCR) methods.

The following data were recorded: age, patient’s preexisting condition (current smoking, diabetes, hypertension, cardiovascular disease, pulmonary disease, and kidney disease), body mass index, ICU length of stay, duration of mechanical ventilation, ventilator-free days at day 28, need for prone position ventilation, and death in the ICU. Initial clinical laboratory workup included a complete blood count and serum biochemical tests. Chest CT scans were performed during the ICU hospitalization. The Simplified Acute Physiology Score (SAPS II) and the Sepsis-Related Organ Failure Assessment (SOFA) score at admission in ICU, at day 7 and 14 days after admission were used to assess severity [7,8].

### 2.2. Aspergillus Detection

Respiratory samples, either bronchial or endotracheal aspirates or bronchoalveolar lavages, were systematic twice weekly and *Aspergillus* detection was performed using culture and real-time quantitative PCR, but GM was not performed to avoid any risk of lab contamination.

Briefly, respiratory samples were first digested using *v*/*v* digestEUR (Eurobio) for 30 min under shaking. Mycological culture were performed after centrifugation of fluidified samples by inoculation of 100–200 µL of pellet on Sabouraud-Chloramphenicol dextrose Agar plates, and incubated for 7 days at 30 °C and 37 °C. Mold identification at genus or species complex level was performed microscopically, and confirmed at species level using MALDI-ToF mass spectrometry (MALDI Biotyper, Bruker France, Marne-la-Vallée, France), after fungal material extraction [9]. Spectrum profiles were then submitted to the Mass Spectrometry Identification (MSI) online database for definitive identification (https://msi.happy-dev.fr/ [10]).

For molecular detection, 200 µL of plain fluidified respiratory sample underwent immediate SARS-CoV-2 inactivation by heating at 56 °C overnight in ATL Lysis buffer (Qiagen, Saint-Quentin Fallavier, France), before DNA extraction using the EZ1 DSP virus kit (Qiagen) on a EZ1 Advanced XL device (Qiagen). Molecular detection of *A. fumigatus* was done using a 28S rDNA *Aspergillus*-targeted PCR, as previously published [11,12].

In case of *Aspergillus* positive culture and/or positive PCR in respiratory samples, additional tests were performed on serum, i.e., detection of GM (Platelia GM *Aspergillus*, Biorad, Marnes-la-Coquette, France), *Aspergillus* PCR and detection of anti-*Aspergillus* antibody by ELISA (Platelia IgG *Aspergillus*, Biorad) and in-house immunoelectrophoresis. Briefly, *Aspergillus* PCR was performed on 1 mL of serum extracted using MagNA Pure 24 Total NA Isolation kit (Roche diagnostics, Meylan, France) on a MagNA Pure 24 device (Roche diagnostics), according to manufacturer recommendations. DNA was eluted in a volume of 50 µL.

### 2.3. Statistical Analysis

Continuous variables were expressed as median (interquartile range, IQR) and compared using the nonparametric Mann–Whitney *U* or Kruskal–Wallis test. Dunn’s correction tests were performed if multiple comparisons were requested. Qualitative data were compared using Chi-square test. Tests were two-sided with significance set at α less than 0.05.

Concordance between categorical results from diagnostic tests was performed using the percent agreement coefficient and Cohen’s kappa coefficient (κ). When comparing quantitative data, an ANOVA test was performed. All data were analyzed with GraphPad Prism 8.4 (GraphPad Software, La Jolla, CA, USA).

## 3. Results

### 3.1. Patient Aspergillus Status

A cohort of 45 COVID-19 intubated and mechanically ventilated patients for ARDS was followed. Patients benefited from a systematic screening for *Aspergillus.* Overall, 211 respiratory samples (culture and PCR) and 32 serum samples (GM detection and *Aspergillus* PCR) were collected. The mean number of respiratory samples until patient discharge from ICU was 3.8 (median = 3).

We categorized these 45 patients according to the AspICU algorithm and propose two alternative classification methods presented in Table 1: the AspICU algorithm associated to PCR results in respiratory and serum samples, and a modified AspICU proposal. Thirty patients did not present any biological criteria of aspergillosis with any of the algorithms. According to the AspICU classification incorporating PCR detection, 15 were classified as having putative aspergillosis because they met all criteria reported by Blot et al., i.e., compatible clinical signs, abnormal thoracic medical imaging on CT scan and positive screening for *Aspergillus* on respiratory samples. However, in this particular context of COVID-19 with all ARDS patients presenting compatible clinical signs and abnormal chest CT imaging in all likelihood lacking specificity, we decided to use a modified AspICU algorithm taking into account blood markers; we classified eight patients as colonized and seven patients with a putative/probable IA (Table 1 and Table 2).

### 3.2. Demographic, Clinical and Biological Characteristics

Demographic, clinical and biological baseline characteristics at admission are detailed in Table 3 and Table S1. Basic demographic characteristics were well-balanced between the three groups of patients (no aspergillosis, *Aspergillus* colonization, putative/probable aspergillosis). Of note, we observed a high proportion (71.1%) of male patients in the study population. Clinical and biological baseline data did not differ among the three groups, except *C*-reactive protein which was higher in the “no aspergillosis” group. Regarding the severity scores at admission, no differences were observed either, among the groups of patients, but SAPS II and SOFA scores at day one tended to be higher in patients with putative invasive aspergillosis.

### 3.3. Concordance of Diagnostic Tools

**Figure 1:**
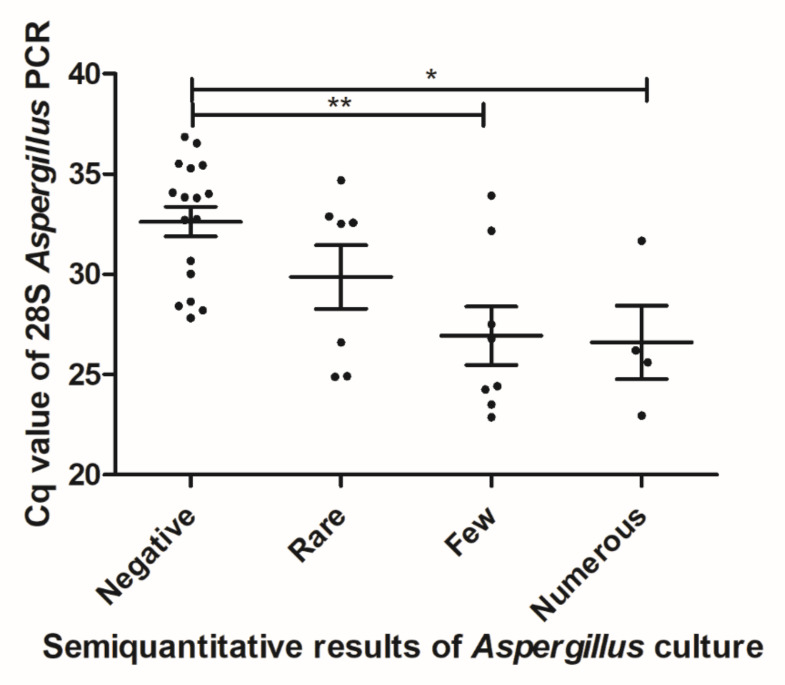
Correlation between molecular and cultural quantification of *Aspergillus* burden in respiratory samples (rare: 1–2 CFU/plate; few: 2–5; numerous: >5). * significantly different with *p* < 0.05. ** significantly different with *p* < 0.01.

Table 4 gathers the results of the techniques used for *Aspergillus* detection. DNA detection by PCR showed the highest sensitivity, with a number of positive respiratory samples near twice higher, compared to the culture. Only one sample grew in culture, whereas PCR was negative, but the species obtained in culture was *A. tubingensis* (*Nigri* complex species), which is theoretically not amplified when using the 28S-targeted PCR specific for *A. fumigatus*. Interestingly, the correlation between cultural and molecular quantification showed a significant difference between the two techniques, with a mean Cq threshold of 32.6 ± 0.7 when cultures were negative, highlighting the higher sensitivity of PCR (Figure 1).

Overall, the concordance coefficient between PCR and culture on respiratory samples was 90.52% with a Cohen’s Kappa coefficient of 0.588. Regarding blood samples, three patients had a positive detection of a systemic biomarker: 3/3 had a positive PCR and 2/3 had a positive GM (Table 5). All three patients had a simultaneous detection of *Aspergillus* in respiratory samples by culture (*n* = 2) and/or PCR (*n* = 3). Overall, the concordance coefficient between PCR and culture on respiratory samples was 93.75% with a Cohen’s Kappa coefficient of 0.632.

### 3.4. Relevance of Various Tests and Categorization of Patients and Outcome

Table 6 presents the classification of the 45 patients using original or modified AspICU algorithms. It appears that using an AspICU algorithm, nine patients were considered as having a putative IA (22% of the cohort). When including PCR, the number of patients with putative IA would increase from 9 to 15 (33%) patients, while most patients might be only colonized because all presented compatible clinical signs and abnormal chest CT scan (Table 5). Regarding *Aspergillus* detection, eight patients had a single detection of fungi using culture and/or PCR in respiratory samples and thus were classified as colonized. One of these patients had a concomitant GM detection in serum (index = 0.551), was not treated and is still alive, thus was considered as a false positive result. Finally, seven (16%) patients presented a heavy burden of *Aspergillus* in the respiratory tract with repeated positive cultures and/or PCR. In order to rule out a chronic colonization before the episode, an anti-*Aspergillus* antibody testing was performed and showed negative results. These patients were classified as putative IA, and three of them could even be considered as probable IA because of a positive biomarker of angioinvasion (serum PCR and/or GM) in agreement with EORTC/MSG classification.

**Figure 2:**
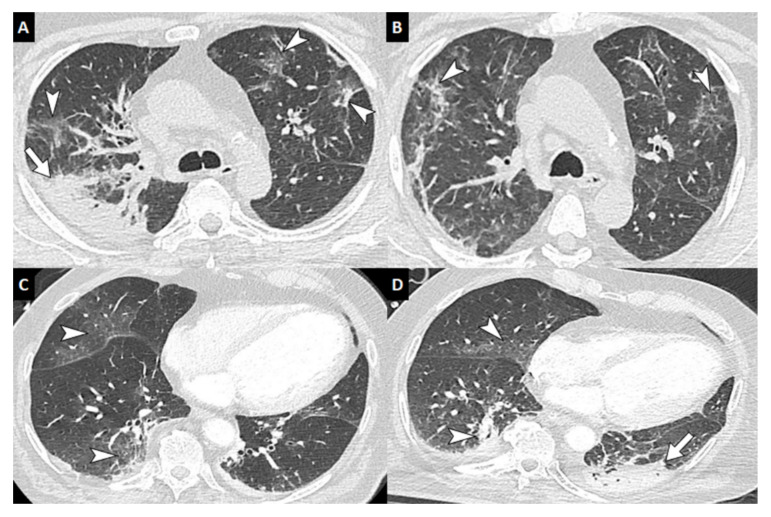
Computed tomography of the chest of patients with COVID-19 with secondary invasive aspergillosis. Unenhanced chest CT in a 59-year-old man with COVID-19 and biological markers of invasive aspergillosis performed at baseline (**A**) and at 12-day follow-up (**B**) showing subpleural ground-glass and reticular opacities presumed to correspond to COVID-19 lesions (arrowheads) as well as a right apical consolidation area presumed to correspond to invasive aspergillosis (arrow). Enhanced chest CT in a 69-year-old man with COVID-19 and biological markers of invasive aspergillosis showing at baseline (**C**) ground-glass opacities (arrowheads), and at 11-day follow-up (**D**) a left postero-basal consolidation presumed to correspond to invasive aspergillosis (arrow). (346-mm field of view, 512 × 512 image matrix, lung window (W1600/L-500 HU)).

Interestingly, following these classification criteria, CT scan abnormalities showed a gradation according to patient group. Diffuse reticular or alveolar opacities were observed in patients classified as probable IA (Figure 2), nodules in half of putative IA, and in colonized patients, only non-specific and hard to interpret signs in the context of COVID-19 infection could be described.

In addition, putative/probable aspergillosis patients appeared more severely ill than patients without aspergillosis, since SOFA score at day seven was significantly higher in this group (*p* = 0.01) with a continuum between no infection, colonization and IA (Table 5). Similarly, the mean ICU length of stay increased significantly from 12 days in patients without aspergillosis to 23 days in colonized patients, and 27 days in putative/probable invasive aspergillosis (*p* = 0.02). All patients with a putative/probable IA were treated either with voriconazole or isavuconazole. Only one colonized patient was treated with voriconazole. Six patients died; there was a trend towards higher mortality in the group of putative/probable IA compared to uninfected patients, although not significant (2/7; 28.6%) versus 4/30 (13.3%), respectively (Table 7).

## 4. Discussion

In France, the global burden of severe fungal infection is estimated at approximately 1,000,000 (1.47%) cases each year [13] and IFD account for a higher risk of mortality in patients with co-morbidities from 9 to 40% [14]. During the COVID-19 pandemic, warning messages considering similarities between Sars-CoV-2 and influenza infections stressed the importance of vigilance towards IFD [15,16]. Local experiences are now published and show high numbers of putative IA [17,18,19,20,21,22].

The diagnosis of IA still remains challenging because of a wide diversity of underlying conditions and growing number of criteria, particularly biological tools [6]. In deeply immunosuppressed patients, such as neutropenic patients, patients under antineoplastic and prolonged corticosteroid therapy or solid organ transplantation, criteria for classification of IFD and notably IA have recently been revised incorporating *Aspergillus* molecular detection [2]. In ICU, the AspICU algorithm published by Blot et al., [3] is a robust and helpful tool for aspergillosis classification but needs to be more evaluated and even updated. In order to address limitations of the various classification definitions for ICU patients, the ongoing FUNgal infections Definitions in ICU patients (FUNDICU) project aims to develop a standard set of definitions for IFD in critically ill patients [5].

The breaking news of SARS-CoV-2 co-infection urges the need for a critical analysis of the criteria of AspICU algorithm. Indeed, COVID-19 patients, particularly ARDS patients with mechanical ventilation, present with compatible clinical signs as depicted by the algorithm (refractory fever, pleuritic chest pain and rub, dyspnea, hemoptysis and worsening respiratory insufficiency, see [3] for full description) and CT-scan signs are hard to interpret because of COVID-19 CT-scan presentation, leading to absence or very poor discrimination between *Aspergillus* colonization and infection [19,23]. As a result, IA during COVID-19 has been reported with a possible overestimated high prevalence (until 30%), as favorable outcomes have been described in patients who did not receive any antifungal treatment.

In order to have a well-balanced patient management, limiting unnecessary and costly antifungal treatments while not neglecting the life-threatening feature of IA, we included *A. fumigatus* PCR as a monitoring tool for fungal detection in both respiratory and blood samples in addition to classical culture and GM approaches but with some restrictions. As expected, PCR allowed detecting *Aspergillus* in much more respiratory samples. We previously showed that PCR improved the detection of *Aspergillus* in BAL, with a particular added value in ICU patients compared to hematology patients [11]. Furthermore, PCR using in-house but also marketed kits is also capable of identifying specific gene mutations associated with azole resistance [11,24]. Besides, the sensitivity of GM detection in blood is less sensitive in ICU than for patients with hematological malignancies [5]. Here, the higher sensitivity of *Aspergillus* detection also incites us to adopt modified criteria for case definition to gain in specificity. Two major changes were introduced to modify the granularity of the classification: (i) the first one is to combine *Aspergillus* detection in respiratory samples and anti-*Aspergillus* antibody testing, to distinguish chronic colonization (positive serology) from acute massive colonization (negative serology) and (ii) the second is to introduce of obvious biomarkers of angioinvasion (serum GM and blood PCR), similar to those of the EORTC/MSG classification [2]. Of note, the combination of positive culture, positive anti-*Aspergillus* antibody testing and positive GM in the context of chronic respiratory diseases characterized a transition step from chronic pulmonary aspergillosis to probable IA [25,26].

Using this refined classification, we were able to categorize our patients in five classes: no infection, colonization, putative IA, probable IA and proven IA (no case of proven IA in the cohort), with a better relevance than the initial AspICU classification, and better specificity than the AspICU + PCR classification. The decision of antifungal treatment onset was taken according to this modified AspICU classification and the outcome observed gives confidence in this patient management. Of course, the limitation of this work is the relatively small number of patients and should be evaluated on larger cohorts in order to correctly analyze the performance of this alternative. A remaining question is also to determine the place of the serum biomarker (1,3)-β-d-glucan in ICU patients, a question that has recently been raised by Honoré et al. [27]

In conclusion, molecular techniques are now key tools for monitoring IFD, particularly IA as recently updated in the EORTC/MSG definitions, but also *Pneumocystis jirovecii* or mucorales infections. Here, we suggest some adaptations of the AspICU clinical algorithm to gain in sensitivity and specificity. Large multicentric data are needed to confirm this proof of concept study.

## Figures and Tables

**Table 1:** Diagnostic criteria of the AspICU clinical algorithm according to Blot et al., 2012, and proposal of a modified AspICU algorithm.

Classification	AspICU According to Blot et al., 2012 [3]	AspICU Algorithm Incorporating PCR	Modified AspICU Algorithm Incorporating PCR, Serology and Angioinvasion Biomarkers
Definition of colonization	*Aspergillus*-positive culture endotracheal aspirate alone	*Aspergillus*-positive culture/PCR endotracheal aspirate alone	*Aspergillus*-positive culture/PCR endotracheal aspirate in one sample, not confirmed on a second sample or using blood biomarker
Definition of putative IA	>1 criterion among: 1. *Aspergillus*-positive culture endotracheal aspirate 2. Compatible clinical signs 3. Abnormal thoracic medical imaging on CT scan or X-ray 4a. Host risk factors Or 4b. Semiquantitative *Aspergillus*-positive culture of BAL fluid + positive direct microscopy	>1 criterion among: 1. *Aspergillus*-positive culture/PCR endotracheal aspirate 2. Compatible clinical signs 3. Abnormal thoracic medical imaging on CT scan or X-ray 4a. Host risk factors Or 4b. Semiquantitative *Aspergillus*-positive culture/PCR of BAL fluid + positive direct microscopy	>1 criterion among: 1. *Aspergillus*-positive culture/PCR endotracheal aspirate in repeated samples with negative anti-*Aspergillus* antibody testing 2. Compatible clinical signs 3. Abnormal thoracic medical imaging on CT scan or X-ray 4a. Host risk factors Or 4b. Semiquantitative *Aspergillus*-positive culture/PCR of BAL fluid + positive direct microscopy
Definition of probable IA	-	-	Putative IA + one positive blood biomarker (GM and/or PCR)
Definition of proven IA	Positive histopathology	Positive histopathology	Positive histopathology

GM: galactomannan.

**Table 2:** Classification of 45 COVID-19 patients with ARDS according to AspICU and to modified AspICU algorithms.

Classification	AspICU According to Blot et al., 2012 [3]	AspICU Algorithm Incorporating PCR	Modified AspICU Algorithm Incorporating PCR, Serology and Angioinvasion Biomarkers
No infection	36	30	30
Colonization	0	0	8
Putative IA	9	15	4
Probable IA	-	-	3
Proven IA	0	0	0

**Table 3:** Demographic characteristics and clinical and biological baseline characteristics.

Demographic Characteristics	All Patients (*n* = 45)	No Aspergillosis (*n* = 30)	*Aspergillus* Colonization (*n* = 8)	Putative/Probable Invasive Aspergillosis (*n* = 7)	*p* Value
Age, years	60 (53–71)	59 (54–68)	53 (51–71)	70 (63–75)	0.14
Sex Men Women	32 (71.1) 13 (28.9)	21 (70) 9 (30)	7 (87.5) 1 (12.5)	4 (57.1) 3 (42.8)	0.42
BMI	27 (24.4–31.4)	27.5 (24.7–32.3)	27 (25.2–30.7)	25.2 (23.2–26.4)	0.99
Current smoking	3 (6.7)	2 (4.4)	0	1 (12.5)	0.54
Coexisting conditions					
Any	31 (68.9)	19 (63)	6 (75)	6 (85.7)	0.47
Diabetes	17 (37.8)	12 (40)	3 (37.5)	2 (28.6)	0.74
Hypertension	15 (33.3)	7 (23.3)	5 (62.5)	3 (42.9)	0.1
Solid cancer	1 (2.2)	1 (3.3)	0	0	0.77
Hemopathy	2 (4.4)	1 (3.3)	0	1 (14.3)	0.54
Cardiovascular disease	3 (6.7)	3 (10)	2 (25)	2 (28.6)	0.34
Chronic obstructive pulmonary disease	0	0	0	0	-
Chronic kidney disease	4 (8.9)	2 (6.7)	1 (12.5)	1 (14.3)	0.83
Temperature (°C)	38 (37–38.9)	37.5 (337–38.4)	38.2 (37.9–39)	38.2 (37.7–38.8)	0.29
Heart rate (/min)	100 (80–110)	94 (80–110)	104 (100–110)	102 (85–119)	0.63
Systolic pressure	94 (87–107)	93 (85–105)	103 (100–109)	90 (82–102)	0.34
White blood cell count (10^9^/L)	9.8 (6.8–12.9)	9.7 (6.9–13)	9.9 (7–10.7)	9.9 (6.7–12.9)	0.97
Neutrophil count (10^9^/L)	7.9 (4.5–10.8)	7 (4.9–10.5)	8.5 (5.2–8.6)	5.6 (3.5–10.4)	0.8
Lymphocyte count (10^9^/L)	0.81 (0.58–1.11)	0.83 (0.53–1.14)	0.7 (0.63–1.1)	0.72 (0.58–0.81)	0.87
Hemoglobin (g/L)	10.8 (9.5–12.5)	10 (9.4–12)	11.8 (10.6–13.6)	11 (10.5–13.6)	0.12
Platelet count (10^9^/L)	264 (194–357)	282 (220–364)	244 (184–347)	162 (129–262)	0.12
Total bilirubin concentration (µmol/L)	8 (5.5–12)	8.5 (6–12)	11 (9–13)	7 (5.5–8)	0.72
Creatinine (µmol/L)	81 (53–162)	71 (51–109)	81 (73–173)	101 (82–184)	0.15
C-reactive protein (CRP) (mg/L)	157 (112–263)	155 (112–265)	112 (102–131)	112 (109–178)	0.03
Ratio of PaO_2_ to F_i_O_2_	152 (100–181)	164 (107–214)	120 (94–214)	136 (72–155)	0.25
SAPS II score on day 1	42 (31–57)	35 (30–58)	42 (21–55)	43 (35–82)	0.55
SOFA score on day 1	7 (2–11)	7 (4–10)	5 (2–10)	9 (2–12)	0.76

Data are presented as median (IQR: interquartiles), *n* (%). *P* values comparing *Aspergillus* colonization, invasive aspergillosis and no aspergillosis groups are tested by Kruskal–Wallis (continuous variables) or Chi-square test (categorical variables). Abbreviations: BMI: Body mass index; SAPS II: Simplified Acute Physiology Score II; SOFA: Sequential Organ Failure Assessment, PaO_2_: arterial oxygen tension.

**Table 4:** Concordance of PCR and cultures on respiratory samples (*n* = 211) to detect the presence of *Aspergillus*.

Respiratory Samples	Positive Culture	Negative Culture	Total
Positive PCR	15	19	34
Negative PCR	1 *	176	177
Total	16	191	211

* positive culture with *Aspergillus tubingensis (Nigri* section).

**Table 5:** Concordance of 28S PCR and galactomannan (GM) in serum samples (*n* = 32).

Serum Samples	Positive GM	Negative GM	Total
Positive PCR	2	1	3
Negative PCR	1	28	29
Total	3	29	32

**Table 6:** Mycological results and classification of 45 COVID-19 patients with ARDS.

Patient	Respiratory Samples		Serum Samples		IA Classification According to		
	*Aspergillus* Positive Culture (nb Samples)	Positive 28S PCR (nb Samples)	GM Index > 0.5 (nb Samples)	Positive 28S PCR (nb Samples)	AspICU (Blot et al., 2012)	AspICU + PCR	Modified AspICU
1	5	5	2	2	putative	putative	probable
2	2	2	1	1	putative	putative	probable
3	0	3	0	1	no infection	putative	probable
4	4	6	0	0	putative	putative	putative
5	4	4	0	0	putative	putative	putative
6	2	5	0	0	putative	putative	putative
7	1	5	0	0	putative	putative	putative
8	1	1	0	0	putative	putative	colonization
9	1	0	1	0	putative	putative	colonization
10	1 *	0	0	0	putative	putative	colonization
11	0	1	0	0	no infection	putative	colonization
12	0	1	0	0	no infection	putative	colonization
13	0	1	0	0	no infection	putative	colonization
14	0	1	0	0	no infection	putative	colonization
15	0	1	0	0	no infection	putative	colonization
16–45	0	0	0	0	no infection	no infection	no infection
Total					9 putative (22%) 36 no infection	15 putative (33%) 30 no infection	3 probable (7%) 4 putative (9%) 8 colonizations (18%) 30 no infection

IA. Invasive aspergillosis, 1 * *Aspergillus tubingensis (Nigri* section).

**Table 7:** Outcomes of patients with COVID-19-associated ARDS according to *Aspergillus* status.

Outcomes	All Patients (*n* = 45)	No Aspergillosis (*n* = 30)	*Aspergillus* Colonization (*n* = 8)	Putative/Probable Invasive Aspergillosis (*n* = 7)	*p* Value
Duration of mechanical ventilation	17 (9–24)	17 (7–24)	18 (10–21)	18 (12–30)	0.66
Ventilator free days at day 28	11 (4–19)	11 (4–21)	10 (7–18)	10 (0–16)	0.64
Prone positioning ventilation	20 (44)	12 (46)	3 (37.5)	5 (71.4)	0.29
SOFA score on day 7	7 (5–11)	6 (5–10)	8 (7–10)	11 (10–12)	0.01
SOFA score on day 14	7 (2–10)	7 (2–9)	3 (1–7)	9 (2–12)	0.2
ICU length of stay	20 (12–27)	12 (11–23)	23 (16–51)	27 (20–36)	0.02
Death in ICU	6 (13.3)	4 (13.3)	0	2 * (28.6)	0.27

Data are presented as median (IQR: interquartiles), *n* (%). *P* values comparing *Aspergillus* colonization, invasive aspergillosis and no aspergillosis groups are tested by Kruskal Wallis (continuous variables) or Chi-square test (categorical variables). Abbreviations: ICU: Intensive Care Unit, SOFA: Sequential Organ Failure Assessment, * 1 putative and 1 probable.

## Supplementary Materials

The following are available online at https://www.mdpi.com/2309-608X/6/3/105/s1, Table S1: Clinical and biological features of the 9 patients classified as putative aspergillosis according to Blot et al., 2012.
