# Supplementary material for: Is the COVID-19 Pandemic a Good Time to Include Aspergillus Molecular Detection to Categorize Aspergillosis in ICU Patients? A Monocentric Experience
Source: J Fungi (Basel). 2020 Jul 10;6(3):105. doi: 10.3390/jof6030105 (PMC7558333; doi:10.3390/jof6030105)
Supplement: Supplementary file 1 [file jof-06-00105-s001.pdf]

Table S1: Clinical and biological features of the 9 patients classified as putative aspergillosis according to Blot et al., 2012.

Patient	Age/Sex	Underlying condition	ICU hospitalization duration (days)	Treatment	Outcome at day 30	*Aspergillus* positive culture (nb resp. samples)	Positive 28S PCR (nb resp. samples)	GM index > 0.5 (nb blood samples)	Positive 28S PCR (nb blood samples)	AspICU (Blot et al., 2012)	Modified AspICU
1	70/M	Cardiomyopathy	34	Isavuconazole 300mg/j	Alive	5	5	2	2	putative	probable
2	79/F	Chronic myelo-monocytic leukemia;hypothyroidia	17	Voriconazole 300mgx2	Died	2	2	1	1	putative	probable
3	59/M	Obesity;hypothyroidia; cardiomyopathy	10	Voriconazole 300mgx2	Alive	0	3	0	1	no infection	probable
4	78/M	Hypertension	25	Voriconazole 300mgx2	Died	4	6	0	0	putative	putative
5	75/M	none	21	Voriconazole 200mgx2	Alive	4	4	0	0	putative	putative
6	73/M	none	65	Voriconazole 200mgx2	Alive	2	5	0	0	putative	putative
7	49/F	Lung transplantation in 2012;hypertension;renal insufficiency	38	Isavuconazole 200mg/j	Alive	1	5	0	0	putative	putative
8	71/M	Obesity;hypertension	11	Voriconazole 200mgx2	Alive	1	1	0	0	putative	colonisation
9	46/F	Obesity;hypothyroidia	9	no treatment	Alive	1	0	1	0	putative	colonisation
10	52/M	Diabetes mellitus; hypothyroidia	16	no treatment	Alive	0	1	0	0	putative	colonisation

Resp.: respiratory.
